# Fabrication of Mucoadhesive Films Containing Pharmaceutical Ionic Liquid and Eudragit Polymer Using Pressure-Assisted Microsyringe-Type 3D Printer for Treating Oral Mucositis

**DOI:** 10.3390/pharmaceutics14091930

**Published:** 2022-09-13

**Authors:** Tatsuaki Tagami, Maya Okamura, Koki Ogawa, Tetsuya Ozeki

**Affiliations:** Drug Delivery and Nano Pharmaceutics, Graduate School of Pharmaceutical Sciences, Nagoya City University, 3-1 Tanabe-dori, Mizuho-ku, Nagoya 467-8603, Japan

**Keywords:** film formulation, oral mucositis, pharmaceutical ionic liquid, pressure-assisted microsyringe (PAM), 3D printing

## Abstract

Oral mucositis in the oral cavity, caused by radiation therapy and chemotherapy, requires personalized care and therapy due to variations in the lesions of patients. In the present study, we fabricated a model of personalized oral film containing an ibuprofen/lidocaine ionic liquid (IL) for patients with oral mucositis using a pressure-assisted microsyringe-type 3D printer at room temperature. The film contained a Eudragit polymer (L100, EPO, or RSPO) to make the film solid, and the printer ink was composed of organo ink (organic solvent to dissolve both drugs and the Eudragit polymer). The viscosity of the printer ink was assessed to investigate its extrudability. The contact angle and the surface tension at the interface between each liquid printer ink and a solid polypropylene sheet were measured to determine the retention of the ink in 3D printing. The physical properties of IL-loaded Eudragit-based dry films were examined by X-ray diffraction and differential scanning calorimetry. Dissolution tests indicated that IL-loaded films containing a Eudragit polymer exhibited different drug release rates in phosphate buffer (pH 6.8; Eudragit L100 > IL alone > Eudragit EPO > Eudragit RSPO). These results provide useful information for the specific fabrication of IL-loaded polymer-based films using organo inks and pressure-assisted microsyringe-type 3D printers.

## 1. Introduction

Oral mucositis is the inflammation of oral mucosa in cancer patients undergoing radiotherapy and chemotherapy, resulting in ulceration due to injury of mitotic cells (mucosal cells) in the oral cavity [1]. The primary symptom of oral mucositis is pain, making it difficult for patients to swallow food and maintain oral hygiene, reducing their quality of life [2]. The side effects of oral mucositis vary greatly for cancer patients. The World Health Organization scale for oral mucositis comprises five grades (grade 0–4) in severity in clinical practice. Thus, personalized therapy fitted to the grade of each patient is required to manage oral mucositis.

Supportive care of oral mucositis is classified into prevention and treatment including pain management [3], and many clinical trials of drugs have been conducted [4]. For example, benzydamine (a nonsteroidal anti-inflammatory drug, NSAID), sucralfate, recombinant growth factor, glutamine, and antibiotics are used for the prevention of oral mucositis, in addition to cryotherapy, and these treatments are combined with mouth wash and systemic administration. An analgesic agent is applied topically before food intake to reduce pain. Anti-inflammatory agents, including NSAIDs, likely reduce the levels of inflammatory cytokines because the pain due to the ulcer is caused by the secretion of inflammatory cytokines such as TNF-α by macrophages, resulting in damage to the mucosal cells. Pediatric patients with solid tumors are systemically administered opioids to relieve pain caused by more severe oral mucositis [5].

Pharmaceutical ionic liquids (ILs) are melted salts that are liquid at room temperature or below 100 °C. Pharmaceutical ILs provide novel options for improving the drug dissolution of poorly water-soluble drugs [6,7], together with typical techniques such as solid dispersion and drug particle pulverization. Pharmaceutical ILs have been investigated for dermal and oral administration, as well as other routes. Lidocaine (Lid)–ibuprofen (Ibu) IL, reported previously [8], was used as a model IL for treating oral mucositis in the present study because this unique IL has analgesic and anti-inflammatory effects, and is promising for managing pain due to oral mucositis.

**Figure 1:**
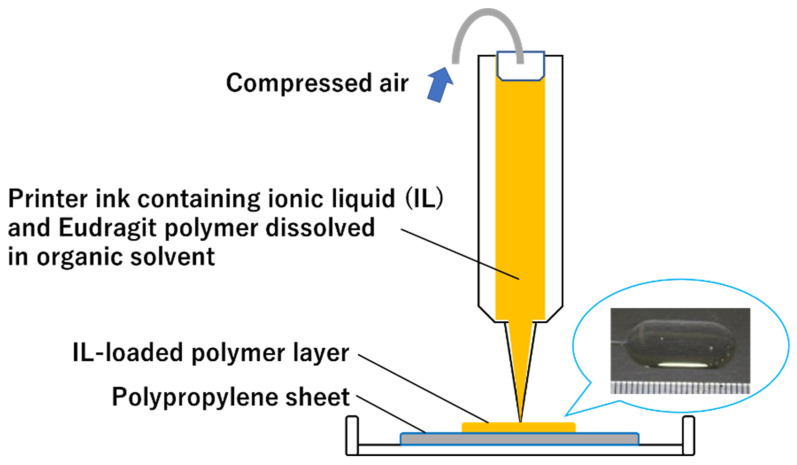
Concept underlying the 3D printing of oral films.

Lid–Ibu IL-loaded films for oral mucositis are fabricated using a pressure-assisted microsyringe (PAM)-type 3D printer capable of semisolid material extrusion, as shown in Figure 1. The 3D printing of drugs is increasing in attention following the approval of 3D printed tablets by the US Food and Drug Administration [9,10]. The pharmaceutical applications of 3D printing have been extended to other dosage forms [11,12]. The unique and highly flexible properties of 3D printing hold promise, providing a paradigm shift in drug design, manufacture, and use [13]. Three-dimensional printing of IL-loaded films has several potential advantages. For example, 3D printing allows the preparation of films with different doses and shapes, allowing personalized dosing. Although the use of ILs containing Lid would make it difficult for patients to eat due to paralysis of the entire mouth, 3D printing of multilayered films, including a supporting layer, would prevent leakage of the IL to unaffected sites in future studies. Furthermore, the viscous and stringy properties of IL films make them difficult to handle in the pharmaceutical industry using a conventional casting method, and also by pharmacists in future clinical settings. In contrast, material extrusion-type 3D printers such as PAM-type 3D printers are useful for producing viscous films, but no proof-of-concept experiments have been reported to date to our knowledge. Thus, here we fabricated IL-loaded films using a 3D printer and characterized and evaluated the films.

## 2. Materials and Methods

### 2.1. Materials

Lid, Ibu, mannitol, and diiodomethane were purchased from Fujifilm Wako Pure Chemical Co. (Osaka, Japan). An Eudragit series (Eudragit L100, EPO, RSPO) was supplied by Evonik Japan Industries Ltd., (Tokyo, Japan). HPMC (Grade, METOLOSE 90SH-15000SR) was supplied by Shin-Etsu Chemical Company, Ltd. (Tokyo, Japan).

### 2.2. Preparation of Printer Ink

As a typical experiment, Ibu (1.03 g, 5 mmol) and Lid (1.17 g, 5 mmol) were dissolved in 10 mL of methanol/acetone (1:1, *v*/*v*) in a screw-top glass vial (vial diameter 28 mm; height 60 mm: As One, Osaka, Japan). The glass vial was placed in a block heater at 60 °C and the organic solvent was evaporated until the intended composition of printer ink was obtained. The compositions of the printer inks are shown in Table 1. To prepare HPMC hydrogel-based ink as a supporting layer, the ink contained 500 mg mannitol, 400 mg HPMC, and 9.1 g water, following the procedure described previously [14].

### 2.3. Viscosity

The viscosities of the printer inks were evaluated using a rotational cone–plate Brookfield viscometer (HB-DV2T, Brookfield, Middleboro, MA, USA), as described previously with modification [15]. Approximately 0.5 mL of printer ink formulation was gently transferred into the cap of the viscometer using an injection syringe. The rotation speed was changed from 0.1 rpm to 200 rpm every 2 min at 25 °C, and the shear stress and viscosity were measured. The viscosity at a shear rate of 1 s^−1^ was acquired by extrapolating the data collected at other shear rates using a linear or quadratic approximation.

### 2.4. Contact Angle

The contact angle of a droplet of printer ink was measured using a B100 contact angle/surface tension meter (ASUMI GIKEN Limited, Tokyo, Japan). A polypropylene sheet (clear holder A4 standard; ASKUL Corporation, Tokyo, Japan) was cut into pieces, placed on the instrument stage, and held in place by adhesive tape. A droplet of printer ink was extruded onto the sheet from a glass syringe (needle, 25G). The contact angle for this droplet (approximately 0.5 µL) was monitored over time, from 0 to 5000 ms, using a Surfscan interfacial analyzer software (ver. 11.5.0.2, ASUMI GIKEN Limited) and photographic images were acquired at each time point.

### 2.5. Surface Tension

The surface tension at the interface between the liquid (printer ink) extruded from a glass syringe (needle, 20G) and air ($\gamma_{L}$) was measured by a B100 contact angle/surface tension meter (ASUMI GIKEN Limited) using the pendant drop method and Surfscan interfacial analyzer software. The surface tension of the printer ink was analyzed by combining the d/D and Young–Laplace methods, which involved the addition of the density data for the printer ink following the manufacturer’s instructions.

The surface free energy (surface tension) at the interface between the solid (polypropylene sheet) and air ($\gamma_{S}$) was acquired using the Owens–Wendt method following the manufacturer’s instructions. Water and diiodomethane were used as probe liquids. We used ‘Young’s equation (Equation (1))’ to obtain the surface free energy (surface tension) at the interface between the solid and liquid ($\gamma_{SL}$). The contact angle was measured for 5000 ms as described in Section 2.4 and used for the calculation.

(1) $$\gamma_{S}=\gamma_{L}cos\theta +\gamma_{SL}$$

### 2.6. 3D Printing and Film Preparation

The 3D printing was conducted using a PAM-type 3D bioprinter (INKREDIBLE; CELLINK, Gothenburg, Sweden), as described previously [14]. Briefly, film-shaped objects (10 mm × 20 mm) were designed using 123D Design 3D CAD software (Autodesk, San Rafael, CA, USA), and the printing conditions were set using Slic3r slicer software (GNU General Public License; printing speed, 10 mm/s; vertical shell, 1; fill density, 100%). These produced G-code data were loaded into the Repetier-Host software (Hot-World GmbH & Co. KG, Willich, Germany). Printer inks prepared as described in Section 2.2 were loaded into a syringe specific for the 3D printer (CELLINK). A polypropylene sheet (ASKUL Corporation) cut to the appropriate size was set on the stage of the 3D printer and taped in place. The 3D printed object containing the IL was dried spontaneously to allow evaporation of the organic solvent. The HPMC-based supporting layer film was generated by freezing the 3D printed object (16 mm × 26 mm) at −80 °C, and then the sample was freeze-dried using a FD-1000 freeze dryer (EYELA, Tokyo, Japan) for 1 day. The ink containing the IL and L100 (formulation B in Table 1) was 3D printed on the freeze-dried HPMC-based film to prepare a multilayer film and then dried.

### 2.7. X-ray Diffraction (XRD) Characterization

A sample of bulk Ibu powder, Lid, excipients, or semisolid or liquid drug formulations was placed on the stage of the XRD equipment (MiniFlex 600, Rigaku Co., Tokyo, Japan) using a micro spatula. The experimental conditions were voltage, 40 kV; and current, 15 mA. The sample was analyzed in the range from 3° to 40°. Samples consisting of Ibu (or Lid) and a polymer (L100, EPO, or RSPO) were obtained by dissolving with an organic solvent and then removing the solvent using a block heater as described in Section 2.2.

### 2.8. Differential Scanning Calorimetry (DSC) Characterization

Each sample (approximately 2–3 mg) prepared as described in Section 2.7 was loaded into an aluminum container (aluminum pan and lid set; Shimadzu, Kyoto, Japan) and crimped shut. An empty container was used as a blank. Each sample was analyzed using a calorimeter (model, DSC-60, Shimadzu, Kyoto, Japan) from 40 to 100 °C at 10 °C/min.

### 2.9. Drug Dissolution Test

Drug dissolution tests were conducted using a general dissolution method (paddle method) following the Japanese Pharmacopoeia. In brief, 900 mL of dissolution medium (phosphate buffer, pH 6.8) was poured into the dissolution vessel, and the medium was maintained at 37 °C under stirring at 50 rpm. Then, the 3D-printed film attached to a rectangle polypropylene sheet (10 mm × 20 mm) was inserted into the dissolution sinker and the sample-loaded sinker was placed in the vessel. At appropriate time points (0, 5, 10, 15, 20, 30, 45, 60, 75, 90, and 120 min), 5 mL of sample was removed and the same volume of dissolution medium was added. The total concentrations of Ibu and Lid were determined by measuring the absorbance at 264 nm using a spectrophotometer (UV-1800, Shimadzu).

## 3. Results and Discussion

Three-dimensional printing technology holds promise for the production of tailored medicines in the pharmaceutical industry. In the current study, we fabricated oral films containing an Ibu–Lid IL by using a PAM-type 3D printer. The films were designed to help alleviate pain in patients with oral mucositis lesions. A PAM-type 3D printer can extrude semisolid and viscous materials. An IL is a melted salt and, thus, should be 3D printable by using this type of 3D printer. However, the IL was not suitable for film formulation due to its flowability and, thus, we solidified the IL by incorporating a Eudragit polymer into the formulation. Three different polymethacrylate-based copolymers (Eudragit polymers L100, EPO, and RSPO) were adopted because Eudragit polymers have unique pH sensitivity and masking effects useful for pharmaceutical excipients [16]. The incorporation of Eudragit polymers was expected to aid film formation but the solidity of the drug formulation hindered its extrusion from the 3D printer nozzle. Thus, we included organic solvent in the printer inks to dissolve both the drugs and the Eudragit polymers, as shown in Table 1. As shown in Figure 1, the “organo inks” were extruded and 3D printed, and the resulting films were obtained by drying the 3D printed objects. The printer inks and resulting films were characterized as described below.

### 3.1. Viscous Properties of Eudragit Polymer-Based Organo Printer Inks

**Figure 2:**
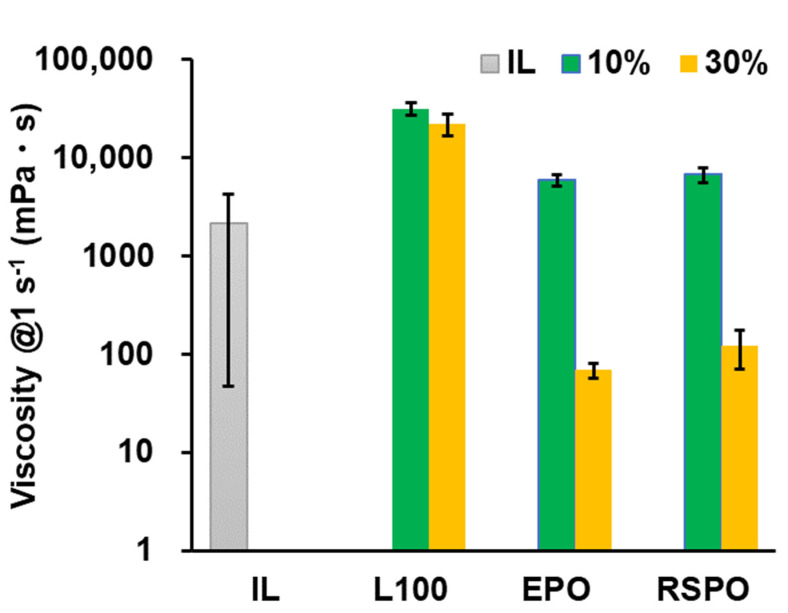
Viscous properties of printer inks containing an IL and different Eudragit polymers. Information on the printer ink formulations (IL, A-F) is shown in Table 1. These data represent the means ± standard deviations (*n* = 3).

We first investigated the viscous properties of printer inks to understand whether an ink was suitable for 3D printing. The extrudability of a printer ink is related to its viscosity and is an important factor in 3D printing using a PAM-type 3D printer. The viscosities of printer inks containing drugs (Ibu and Lid), organic solvent, and Eudragit polymers are shown in Figure 2. Eudragit L100-containing ink formulations exhibited high viscosity compared to Eudragit EPO-containing and Eudragit RSPO-containing formulations, likely due to the molecular weight of Eudragit (Eudragit L100, 125,000; Eudragit EPO, 47,000; Eudragit RSPO, 35,000 [17]). The present results suggest that the structural viscosity of polymers in an organic solvent is a key determinant of viscosity. The viscosity of the IL was intermediate among the compared ink formulations. Panić et al. reported the viscosity profiles for Ibu–Lid ILs as following Newtonian behavior [18], supporting our present results. Inks containing different amounts of organic solvent (10% printer ink formulation A, C, and E; 30% printer ink formulation B, D, and F) revealed that inks with a high amount of organic solvent had lower viscosity. Interestingly, the Eudragit L100-containing formulations A and B had smaller viscosities despite the different amounts of organic solvent. Thus, the addition of Eudragit L100 to printer ink resulted in less sensitivity to the amount of organic solvent under the present experimental conditions. The present range of viscosities was 3D-printable in our study, and ink formulations IL, B, D, and F allowed successful 3D printing.

In our previous study, HPMC-based hydrogel and HPMC/gelatin-based hydrogel were used as printer inks to generate films and gummy formulations [14,15,19]. The properties of paste, hydrogel, and hydro-alcoholic gel have been reported, but to our knowledge, those of polymer-based organo inks for material extrusion-type 3D printers have not been studied. We, therefore, evaluated the contact angle and surface tension of the organo inks.

### 3.2. Contact Angle and Surface Tension of Eudragit Polymer-Based Organo Inks

**Figure 3:**
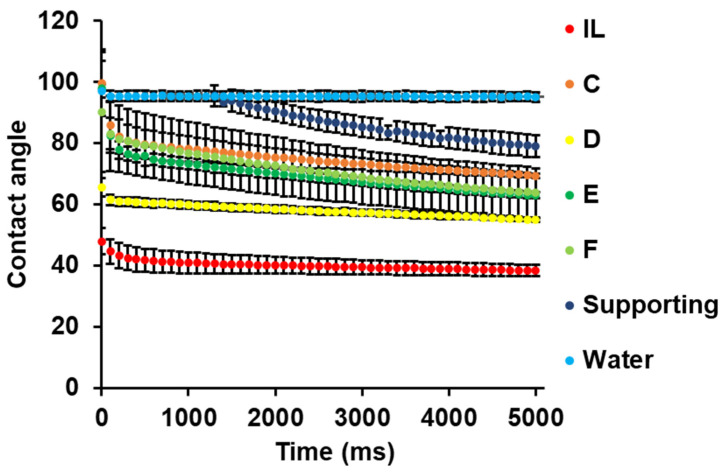
Contact angles of printer inks containing an IL and different Eudragit polymers. Information on the printer ink formulations is shown in Table 1 and in Section 2. These data represent the means ± standard deviations (*n* = 3).

To determine the properties of printer inks, we investigated the change in contact angle for each printer ink with time (Figure 3). We speculated that printer inks with optimal contact angles could form film-like objects on the stage by 3D printing. If the contact angle of a printer ink is high (e.g., 90°<), the 3D printed film object can become spherical with time, whereas if the contact angle is low, the 3D printed film object can easily collapse and spread on the surface of another material due to wettability. A droplet of printer ink was placed on a polypropylene sheet (the same material as placed on the stage of the 3D printer). The contact angles followed the order: water > supporting layer > formulation C > formulation E and F > formulation D >> IL. This trend was remarkably different from that for the viscosity (Figure 2). Viscosity might have affected the change with time in the contact angle, as the contact angles of printer inks with lower viscosity (formulation D and F) drastically dropped within 100 ms. EPO-incorporated printer ink and RSPO-incorporated printer ink exhibited different contact angles. This was probably due to the functional group in the chemical structure of the Eudragit series, although further studies regarding the interaction between printer ink and other types of polymer sheets are required to clarify the underlying mechanism. The IL exhibited the lowest contact angle (≈40°) among the samples, indicating that the IL might have easily spread after 3D printing. We could not measure the contact angle of L100-containing printer inks (formulation A and B) due to their high viscosity and thread-forming properties.

The surface tension of each printer ink was measured (Supplementary Table S1). $\gamma_{S}$, the surface free energy (surface tension) at the interface between the solid (polypropylene sheet) and air, was acquired (36.42 mJ/m^2^, constant). $\gamma_{L}$, the surface tension at the interface between the liquid (IL-loaded printer ink) and air, was similar (32.7–35.4 mN/m). The $\gamma_{L}\;$ for HPMC-based ink used as the supporting layer was lower than that for water, probably because HPMC molecules affected the surface tension of water molecules at the interface. These results suggest that the surface tension of a printer ink is highly dependent on the type of material (polymers, organic solvents, and drugs) in the ink. We could not determine the surface tension of formulation A.

We obtained the value of $\gamma_{SL}$, the surface free energy (surface tension) at the interface between the solid and liquid (Supplementary Table S1). This surface free energy is directly related to the retention of printer ink on the surface of a propylene sheet. It was noteworthy that the order of contact angles of inks containing an IL was the same as that of $\gamma_{SL}$ (formulation C > formulation E and F > formulation D > IL, Figure 3). Since $\gamma_{S}$ and $\gamma_{L}$ were constant and similar, the contact angle of a printer ink could be used to estimate $\gamma_{SL}$ through Young’s Equation (1). The $\gamma_{SL}$ of the IL was much smaller than that of other IL-containing printer inks, so the IL might have had relatively high spreadability. In contrast, HPMC-based hydrogel had a lower surface tension and higher contact angle than printer inks containing an IL, and an overall larger $\gamma_{SL}$, resulting in good 3D printing; a similar composition ink was used previously [14]. Contact angles may, thus, help clarify the printability of a film formulation on a sheet when inks with similar components are compared.

### 3.3. 3D Printing and the Resulting IL-Loaded Polymer Films

The appearance of the printer inks is shown in Table 2. The ink formulations were clear due to complete dissolution of the ingredients. Printability was confirmed, and the appearance of the 3D printed film-like objects after drying are shown in Table 2. The formulation IL was extrudable, but its syrup-like properties and flowability slightly hindered retention of the film shape. In contrast, printer ink formulations containing Eudragit polymers, especially formulation B, were extrudable and formed film-like shapes after drying.

We obtained films containing 20–40 mg Ibu and Lid, but the weight of the resulting 3D-printed objects varied slightly because the different viscosities of the printer inks affected the amount extruded. Although further improvement is necessary, the extruded amount can be predicted from the viscosity. Elbadawi et al. constructed a machine learning model to predict printability and drug dissolution from viscosity [20].

### 3.4. Physical Characteristics of IL-Loaded Polymer Films

**Figure 4:**
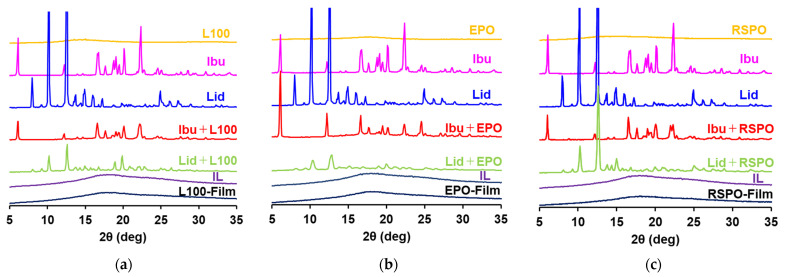
XRD analysis of the components of printer ink, the combination of drug and Eudragit polymers, ILs, and IL-loaded films. (**a**) XRD analysis of L100-containing samples. (**b**) XRD analysis of EPO-containing samples. (**c**) XRD analysis of RSPO-containing samples.

XRD measurements were conducted to determine the physical properties of the IL-loaded films (Figure 4). Bulk Ibu and Lid powder had high crystallinity, while Eudragit polymer (L100, EPO, RSPO) powders were in an amorphous state. IL-loaded Eudragit films showed no peaks, strongly suggesting that both drugs were present as either amorphous or IL forms in the Eudragit polymer films. Most of Ibu–Eudragit and Lid–Eudragit samples were in the solid state, without flexibility at room temperature under our experimental conditions. This state may not be suitable for film formulation, which requires flexibility. In contrast, the IL-loaded Eudragit films were flexible. These results indicate that most drugs can presumably adopt an IL form, although we do not conclude that the Eudragit film formulations prepared in the current study are present in ionic liquids or eutectic mixtures. The presence of an IL in a film acts as a plasticizer in the current film formulation, allowing the formulation to be applied to a diseased site for practical use.

The Ibu–Eudragit and Lid–Eudragit samples provided relatively weak peaks compared to bulk Ibu and Lid powders. Each drug was presumably partially amorphous since Eudragit polymers are used to prepare solid dispersions [17]. These samples were clear at first, while several crystallizations were finally observed during storage. For example, Liu et al. reported that extrudate containing 30% Lid and 70% L100 prepared by hot-melt extrusion was amorphous [21], and Patwardhan et al. reported that extrudate containing 50% Ibu and 50% EPO prepared by hot-melt extrusion was amorphous [22].

**Figure 5:**
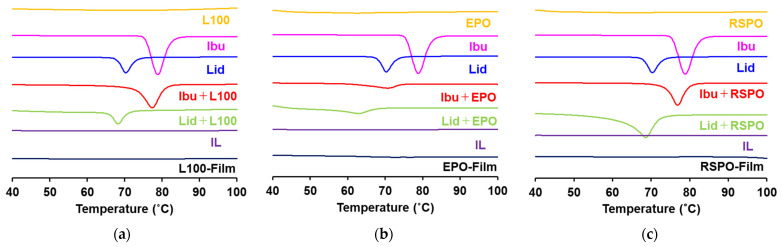
DSC analysis of the components of printer ink, the combination of drug and Eudragit polymers, ILs, and IL-loaded films. (**a**) DSC analysis of L100-containing samples. (**b**) DSC analysis of EPO-containing samples. (**c**) DSC analysis of RSPO-containing samples.

The results of the DSC analysis confirmed those of the XRD analysis (Figure 5). Bulk Ibu and Lid powders showed sharp endothermic peaks, while Eudragit polymer powders had relatively broad peaks at approximately 200 °C (data not shown). In contrast, IL-loaded Eudragit films lacked the endothermic peaks of the Ibu- and Lid-like IL sample. The results suggest that both drugs are present in the amorphous and IL form in Eudragit polymers. The peaks obtained for the Ibu–Eudragit and Lid–Eudragit samples shifted to lower temperature compared to Ibu or Lid alone, due to being eutectic mixtures.

### 3.5. Drug Release from 3D Printed Films Composed of Different Eudragits and ILs

**Figure 6:**
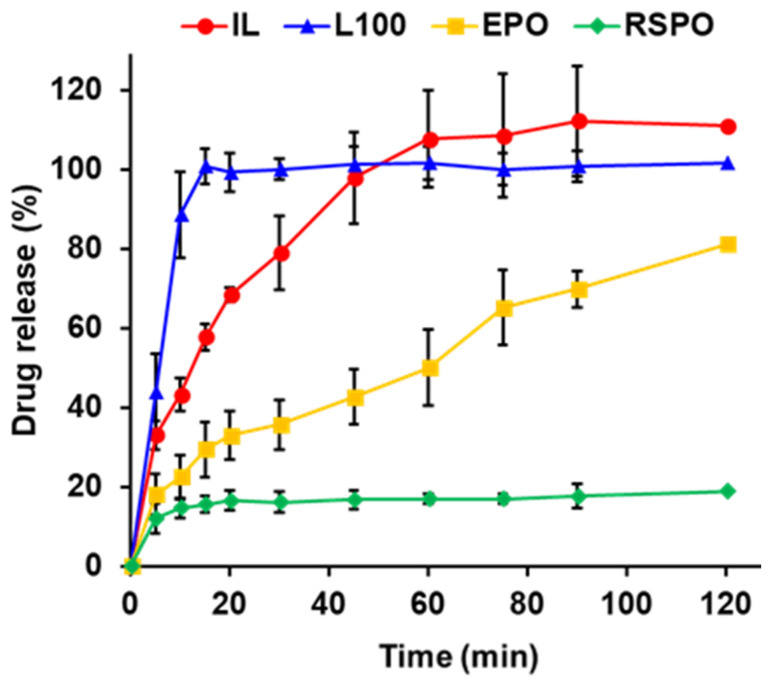
Drug release from 3D-printed films comprising different Eudragit polymers and an IL. Details of the drug dissolution test and the drug formulations used are described in Section 2 and Table 1, respectively. These data represent the means ± standard deviations (*n* = 3).

In this test, drug release from one side of the film was investigated by attaching the film onto a polypropylene sheet to simulate localized drug release into oral mucositis (Figure 6). The order of drug release was L100-based film > IL alone > EPO-based film > RSPO-based film. L100 is pH-sensitive and water-soluble at near neutral pH (pH 6 <) [17]. In neutral dissolution medium, L100-based film released the drug quickly and the release was faster than that for the IL alone. These results suggest that drug release from L100-based film is effective under physiological conditions, such as oral mucositis in the oral cavity of cancer patients. The EPO-based film exhibited sustained drug release compared to the IL alone, and more than 80% of the drug was released after 120 min. EPO is pH-sensitive at acidic pH and is soluble. Drug release at neutral conditions was minimal in the current study, suggesting that EPO is an option for sustained drug release. The RSPO-based film did not exhibit effective drug release (<20%) after 120 min. RSPO is a poorly water-soluble polymer and has been studied as a tablet coating for controlled release [17]. We observed that the EPO-based and RSPO-based films were still remaining on the sheets after 120 min of dissolution testing.

### 3.6. Fabrication of Multilayer Films Using a PAM-Type 3D Printer

**Figure 7:**
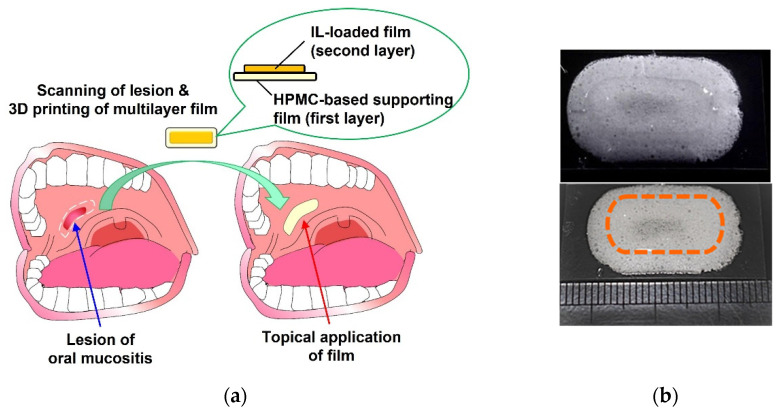
(**a**) Scheme describing the application of a 3D printed film to an oral mucositis site. (**b**) Appearance of a multilayer film comprising an IL-loaded layer and a HPMC-based supporting layer. Information on the printer ink formulation (formulation B) and the HPMC-based ink is shown in Table 1 and in Section 2.

We fabricated multilayer films using a PAM-type 3D printer. A HPMC-based supporting layer was 3D printed and freeze-dried, then printer ink containing an IL and L100 was laminated on top and dried. As shown in Figure 7a, a multilayer film including a supporting layer is useful because it can ensure drug delivery only to the pathological site. The leakage of IL containing Lid can minimize paralysis of the oral cavity, which can greatly help patients stay without pain and, thus, increase their quality of life. The 3D CAD data of a pathological site can be 3D scanned using a 3D scanner and/or computed tomography so that a bone scaffold can be 3D printed and fabricated in bone tissue engineering [23,24]. A multilayered film consisting of a freeze-dried HPMC-based supporting film and an IL-loaded L100 film was fabricated successfully (Figure 7b). HPMC-based film has mucoadhesive properties and feels like cotton, suggesting that the film is useful for application in the oral cavity [14]. Although the data in this section were preliminary, we think that the 3D printing of multilayer films with various shapes is useful for tailored medicines for patients.

## 4. Conclusions

In conclusion, we herein reported IL-loaded polymer-based films prepared using a PAM-type 3D printer. Assessing the viscosity of organo printer inks is useful for determining their extrudability using a PAM-type 3D printer, and assessing the contact angle and $\gamma_{SL}$ of the inks can clarify the interaction of the inks with polymer sheets in 3D printing. The incorporation of a series of Eudragit polymers affects the properties of printer inks and of printed objects, including their drug release behavior. This technology could be applicable for not only other pharmaceutical IL formulations, but also for topical applications used in hospital settings. Although further experiments are necessary, this study provided useful information for the application of 3D printing to personalized therapy for patients with pain in their oral cavity caused by, for example, oral mucositis.

## Figures and Tables

**Table 1:** Compositions of ink formulations for 3D printing.

Formulation	Ibu (g)	Lid (g)	Eudragit L100 (g)	Eudragit EPO (g)	Eudragit RSPO (g)	Organic Solvent (g)	Used for 3D Printing
IL	1.03	1.17	-	-	-	-	✓
A	1.03	1.17	0.55	-	-	0.275	
B	1.03	1.17	0.55	-	-	0.825	✓
C	1.03	1.17	-	0.55	-	0.275	
D	1.03	1.17	-	0.55	-	0.825	✓
E	1.03	1.17	-	-	0.55	0.275	
F	1.03	1.17	-	-	0.55	0.825	✓

**Table 2:** Appearance of printer ink formulations and IL-loaded films after 3D printing and drying. The weights of the resulting films and estimated weight of Ibu and Lid in the films are shown. Information on the printer inks is shown in Table 1. These data represent the means ± standard deviations (*n* = 3).

	Formulation IL	Formulation B (L100-film)	Formulation D (EPO-film)	Formulation F (RSPO-film)
Appearance of printer ink	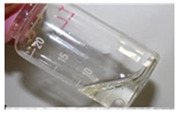	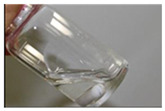	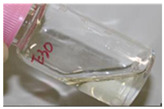	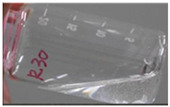
Appearance of IL-loaded film	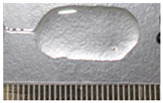	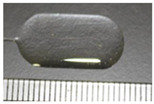	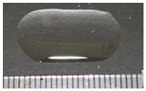	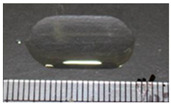
Weight of film (mg)	43.8 ± 2.7	57.1 ± 2.5	59.2 ± 5.3	96.2 ± 2.3
Ibu (mg)	20.5 ± 1.3	21.4 ± 0.93	22.2 ± 2.0	36.0 ± 0.85
Lid (mg)	23.3 ± 1.4	24.3 ± 1.1	25.2 ± 2.3	40.9 ± 0.96

## Data Availability

Not applicable.

## Supplementary Materials

The following supporting information can be downloaded at: https://www.mdpi.com/article/10.3390/pharmaceutics14091930/s1, Table S1: surface tension and density of IL-loaded printer inks containing different Eudragit polymers. Information on the printer inks is shown in Table 1 and in Section 2. These data on surface tension represent the means ± standard deviations of five measurements. ND *: Not determined. ND ^†^: No data available.
