# Supplementary material for: Fabrication of Mucoadhesive Films Containing Pharmaceutical Ionic Liquid and Eudragit Polymer Using Pressure-Assisted Microsyringe-Type 3D Printer for Treating Oral Mucositis
Source: Pharmaceutics. 2022 Sep 13;14(9):1930. doi: 10.3390/pharmaceutics14091930 (PMC9505851; doi:10.3390/pharmaceutics14091930)
Supplement: Supplementary file 1 [file pharmaceutics-14-01930-s001.zip › pharmaceutics-1894899-supplementary.pdf]

Table S1. Surface tension, density, and γ_{SL} of IL-loaded printer inks containing different Eudragit polymers. Information on the printer inks is shown in Table 1 and in Materials and Methods. The data on surface tension represent the mean ± standard deviation of five measurements. ND *: Not determined. ND †: No data available.

Formulation	IL	A	B	C	D	E	F	Supporting Layer	Water
**Surface Tension （mN/m）**	33.3 ± 0.6	ND *	34.9 ± 0.6	35.4 ± 3.2	32.9 ± 0.6	34.8 ± 0.5	32.7 ± 0.1	19.8 ± 5.8	70.7 ± 0.8
**Density (g/cm³)**	1.01766	1.02419	1.03106	1.05111	0.99193	1.05837	1.00968	1.02592	0.99795
**γ_{SL}(mJ/m²)**	7.9	ND †	ND †	24.0	17.5	20.6	22.0	32.6	42.6
